# Enhancing Inclusive Teaching in China: Examining the Effects of Principal Transformational Leadership, Teachers’ Inclusive Role Identity, and Efficacy

**DOI:** 10.3390/bs14030175

**Published:** 2024-02-23

**Authors:** Dongsheng Wang, Liang Huang, Xianhan Huang, Meng Deng, Wanying Zhang

**Affiliations:** 1 Faculty of Education, Beijing Normal University, Beijing 100875, China; wangdongsheng@mail.bnu.edu.cn (D.W.); wanyzhang@mail.bnu.edu.cn (W.Z.); 2 Faculty of Education, Northwest Normal University, Lanzhou 730070, China; 3 Department of Public Administration, Southeast University, Nanjing 211189, China; 4 Department of Curriculum and Instruction, The Education University of Hong Kong, Hong Kong 999077, China; xhhuang@eduhk.hk; 5 Special Education Department, Faculty of Education, East China Normal University, Shanghai 200062, China; mdeng@ed.ecnu.edu.cn

**Keywords:** inclusive education, transformational leadership, inclusive role identity, teacher efficacy, inclusive teaching behaviour

## Abstract

This research examined the effects of principal transformational leadership on teachers’ inclusive teaching behaviour, with a particular inquiry into the mediating effects of teachers’ inclusive role identity and efficacy for inclusive practice, as informed by identity theory and social cognitive theory. Structural equation modelling with bootstrapping estimation was conducted using data from 712 teachers delivering inclusive teaching in primary or secondary schools in China. The results revealed the sequentially mediating mechanisms of teachers’ inclusive role identity and efficacy underlying the principal transformational leadership effects on teachers’ inclusive teaching behaviour. Research implications are also discussed.

## 1. Introduction

Since the Salamanca Statement [1], providing high-quality and inclusive education for all students has become a prioritised goal for educational systems across the globe. Many countries worldwide have implemented inclusive education (IE) reforms to increase inclusion and equity in schools [2,3]. Despite significant efforts to promote IE, education systems continue to face challenges in fulfilling their obligations and improving inclusive practices [4,5].

Teachers are considered one of the most pivotal agents in implementing IE reforms [6]. Teachers’ inclusive teaching behaviour (TITB) means that teachers respond to learners’ diversity needs and support the participation and achievement of all students in class [7]. It is evident that TITB is significantly related to students’ academic performance in both specific subjects (e.g., reading and mathematics) and overall school performance in general [8,9,10,11]. In light of its paramount importance in school reforms aiming at promoting inclusion, extensive research has been conducted to examine influencing factors of TITB, such as efficacy beliefs, behavioural intention, and attitudes towards inclusive education [12,13]. Beyond these individual-level influences, increasing research attention has been devoted to school-level contextual factors such as inclusive climate and institutional support [14,15]. Although some antecedents have been identified, it remains empirically unclear how teachers’ inclusive teaching behaviours are linked to principals’ leadership practices.

Among various school-level influences in relation to teachers’ teaching practice, principal transformational leadership (PTL) has represented particular significance [16]. As a specific leadership model that emphasises leaders empowering followers to achieve meaningful change through inspirational motivation and capacity development [17], transformational leadership has been recognised as particularly influential in stimulating organisational commitment, fostering behavioural changes, and promoting high-standard performance and productivity [18,19,20]. However, in the context of IE reforms, despite the salient potential of PTL to reshape mainstream classroom teaching to cater to the needs of diverse learners as recognised in policy, research, and practice [21,22], few empirical studies have examined the effects of PTL on TITB. Even fewer still go further to elucidate the mechanism as to how PTL shapes TITB.

Teachers’ inclusive role identity (TIRI) and efficacy for inclusive practice (TEIP) were examined as two teacher-level factors that mediated the PTL effects on TITB. On the one hand, identity theory [23,24] emphasises that the way teachers perceive and define themselves within their professional roles, as well as their interactions with significant others, has a profound influence on their beliefs and actions related to daily teaching behaviours [25]. On the other hand, teacher efficacy, as informed by social cognitive theory [26], has been consistently identified as a crucial mechanism through which the influences of PTL are transmitted and impact teachers’ instructional practices. This is especially evident in challenging and demanding contexts [16,27], such as inclusive teaching situations. Moreover, empirical literature in general education and social psychology has demonstrated that individuals’ role identity may positively affect their efficacy [28], which indicates a sequential mediation of these two teacher-level psychosocial influences. However, no empirical studies, to our knowledge, have examined the effects of PTL on TITB through the sequential mediation of TIRI and TEIP.

To address the noticeable research gaps and to inform effective principal leadership practices, professional training programmes, and teacher development strategies to promote TITB in the context of IE reforms, the present study was conducted with a sample of Chinese teachers teaching inclusively in primary or secondary schools. Specifically, the following questions were proposed to guide the study:

1. To what extent does principal transformational leadership influence teachers’ inclusive teaching behaviour?
2. Do teachers’ inclusive role identity and efficacy independently mediate the effects of principal transformational leadership on teachers’ inclusive teaching behaviour?
3. Do teachers’ inclusive role identity and efficacy sequentially mediate the effects of principal transformational leadership on teachers’ inclusive teaching behaviour?

## 2. Literature Review

### 2.1. Principal Transformational Leadership and Teachers’ Inclusive Teaching Behaviour

Principal transformational leadership refers to principals inspiring and motivating teachers to achieve positive change and attain professional progress in accordance with individualised needs [29,30]. Transformational leadership emphasises shared values and vision, attributes importance to symbolic behaviour, and empowers teachers for meaningful teaching to promote change and innovation [31]. According to Leithwood and Jantzi’s theory [19], three core dimensions contribute to the construct of school transformational leadership: setting directions through building a shared vision, developing people, and redesigning the organisation. In this research, PTL is conceptualised as a construct in which principals promote inclusive education in ways of setting direction for inclusion, supporting teachers for inclusive practice, and redesigning the organisation to enhance teacher involvement towards inclusive education [21,22,32].

Studies have found that principal transformational leadership is positively related to teachers’ teaching behaviours and work performance [16,33,34]. PTL can pose significant influences on teachers’ teaching practice through such leadership behaviours as defining inspirational goals and persuasive values for teaching excellence, stimulating teachers’ competences that foster innovation, and creating empowering and facilitative structures that spur teaching change [35]. In the context of IE, despite few studies examining the effect of PTL on teachers’ inclusive teaching behaviour (TITB), some qualitative research indicated that in schools where principals conduct leadership practices in transformational ways, such as setting inclusive visions, developing inclusive methods of teaching, and fostering supportive structures, teachers tend to integrate inclusive strategies (e.g., individualised teaching planning, differentiated instruction, and adaptive teaching) into classroom teaching for students with diverse learning needs [14,36,37,38,39].Therefore, we postulate that principal transformational leadership in prompting inclusive education may positively influence teachers’ inclusive teaching behaviour.

**H1.**  *PTL has a significantly positive influence on TBTI*.

### 2.2. Teachers’ Inclusive Role Identity

Teachers’ inclusive role identity (TIRI) has theoretical origins in identity theory. Role identity was defined as the way individuals prefer to see themselves and be seen by others while being and acting as occupants of specific social positions or roles [40]. In reference to various models of role identity, such as creative [41], founder [42], and volunteer role identity [43], we conceptualised teachers’ inclusive role identity as the way teachers like to perceive themselves or be perceived by others as individuals prioritising and valuing inclusion and related professional roles.

While research evidence remains scarce as to how teachers’ inclusive role identity influences teachers’ inclusive teaching behaviour, identity theory postulates inherent connections between them [25,44]. Identity theory mentions that individuals are motivated to maintain consistency between their identity meaning and behaviour [28,45]. When teachers’ work role is closely tied to their identity, they are more inclined to behave in accordance with the expectations associated with the role [45,46]. Empirical studies have shown that the higher the salience of a role identity, the greater the probability of behavioural choices in accord with the expectations attached to that role identity [47,48]. In this sense, teachers who develop an inclusive role identity as inclusive educators can be motivated by the internal awareness of being empowered agents for inclusion [24,49]. This motivation may compel them to strive for improvement in their inclusive teaching behaviours.

Identity theory also suggests that PTL may shape teachers’ inclusive role identity. According to the theory, social information from significant others represents the primary and fundamental source for the formulation of individuals’ role identity [47]. Within the context of IE, PTL can serve as a significant catalyst for stimulating teachers’ inclusive role identity. Setting high expectations for inclusion, supporting inclusive change, and nurturing teacher capacities will serve as means of conveying persuasive cues and normative expectations [22,50]. Qualitative research also found that principals’ commitment and facilitation of IE may help improve teachers’ inclusive role identity. When principals advocate for inclusive change and prioritise a vision for IE, teachers will be more motivated to embrace the presence of children with special needs and adapt their teaching behaviours accordingly [51]. Based on the identity theory and empirical studies, it is possible to hypothesise as follows:

**H2.**  *Teachers’ inclusive role identity positively mediates the influences of PTL on TITB*.

### 2.3. Teacher Efficacy for Inclusive Practice

Self-efficacy is defined as a judgement of the capability to execute a given type of performance [26]. As teacher efficacy is considered context-specific [52], teacher efficacy for inclusive practice (TEIP) is conceptualised as teachers’ beliefs in their abilities and skills to implement inclusive teaching practice. According to social cognitive theory [26], teachers’ inclusive teaching behaviours can be greatly influenced by their sense of efficacy. Inclusive teaching requires that teachers respond to the challenges of valuing each student’s progress, employing diverse teaching approaches, and continuously reflecting upon inclusive practices [53]. This highlights the critical importance of teacher efficacy as a psychological strength that enables inclusive, high-quality teaching for all students [10]. The existing literature has shown consistent evidence that teachers with a strong inclusive teaching efficacy are more open to diversity and inclusion, and demonstrate more persistence and resilience in overcoming obstacles to implement inclusive practices [12,54,55].

Social cognitive theory also suggests that principal transformational leadership as a school contextual factor influences teacher efficacy for inclusive practice [56]. Geijsel, F. P., P. J. C. Sleegers, R. D. Stoel and M. L. Kruger [57] validated and elucidated this relationship by analysing three potent traits of transformational leaders. First, principals as transformational leaders empower their followers to achieve goals by clearly articulating the vision, explaining how to attain it, expressing confidence and optimism, and actively communicating norms and beliefs to their followers. As a result, individuals may experience an increased sense of assurance in their ability to attain the vision. Second, transformational leaders enhance teachers’ problem-solving abilities and deepen their understanding of their own values, thereby increasing their teaching efficacy. Third, modelling inclusive practices from transformational leaders will boost teachers’ confidence in overcoming the challenges of inclusive teaching, as Bandura, A. [26] suggested. As informed by social cognitive theory and extensive literature demonstrating the positive effects of PTL on teachers’ teaching efficacy and subsequent teaching practices [12,16,56], it is reasonable to hypothesise as follows:

**H3.**  *Teacher efficacy for inclusive practice positively mediates the influences of PTL on TITB*.

### 2.4. Sequential Mediation of Teachers’ Inclusive Role Identity and Efficacy for Inclusive Practice

The existing literature has revealed a potential relationship between teachers’ inclusive role identity and efficacy. Burke, P. J. and D. C. Reitzes [23], in their theoretical extrapolation, proposed that efficacy is derived from role identity, and in turn, role identity promotes the development of efficacy development. When individuals seek to verify and confirm their identity, they often dedicate more time and effort to participating in activities that are directly related to their identity [28,46]. This increased engagement and the consequent positive experiences contribute to a sense of efficacy [26]. Moreover, emerging research in the field of psychology and general education also provides empirical clues [28,58,59]. Given that teachers’ inclusive role identity and efficacy for inclusive practice are deeply shaped by principal transformational leadership and may interact to influence teachers’ inclusive teaching behaviour [60], the following hypothesis can be proposed:

**H4.**  *Teachers’ inclusive role identity and efficacy for inclusive practice sequentially mediate the influences of PTL on TITB*.

### 2.5. The Hypothesised Model of Study

**Figure 1:**
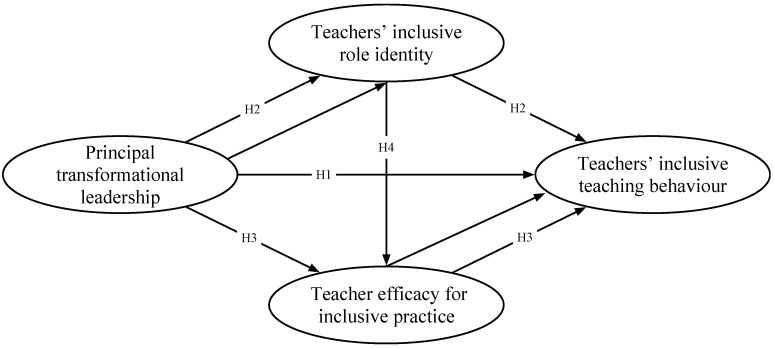
Hypothesised model of the study.

Based on the literature review, this study will examine the mechanism of how principal transformational leadership influences teachers’ inclusive teaching behaviour. The hypothesised model of the study is proposed in Figure 1.

## 3. Research Context

In China, inclusive education is implemented via the initiative Learning in Regular Classrooms (LRC, sui ban jiu du), an indigenous practice for educating children with disabilities in regular classrooms within regular schools. In the 1980s, China initiated the LRC policy to ensure that children with disabilities previously denied access to education receive compulsory school education [61]. In line with the global trend of inclusive education, China has expanded the rationale of LRC to achieve equitable and high-quality education and positioned inclusive education as an indispensable approach to educational modernisation. In 2019, the Chinese government introduced a monumental policy document, China’s Education Modernisation 2035, to achieve a high-quality education, stressing ‘inclusive development’ as one of the fundamental educational concepts and ‘comprehensively promoting inclusive education’ as an essential objective towards 2035 [62].

However, LRC has been long criticised as ‘sitting in regular classrooms’, indicating that students with disabilities are included only physically rather than academically and socially [63]. Two critical factors regarding schools’ administration and teachers’ teaching practices have been mentioned in practice and theory. On the one hand, effective school leadership for IE is crucial to systematic school reforms promoting inclusive school culture, organisational structure, adaptive support, curriculum, and instruction, etc. [15]. On the other hand, due to the traditional dichotomy of special and general education in China, many teachers in regular schools lack role identification in teaching children with disability in their classrooms, which impedes them from implementing inclusive teaching behaviours to cater to diverse educational needs. 

As China strives towards fostering inclusive education comprehensively, the role of principals’ transformational leadership practices becomes crucial. Considering the collectivist and high-power distance culture in Chinese schools [64,65], the mechanism of PTL is probably different, as it may have a more powerful influence on teachers’ work behaviours.

## 4. Method

### 4.1. Participants and Procedures

The participating teachers in this research were from regular primary and secondary schools in China’s Gansu province and Ningxia Hui Autonomous Region. They were invited via email to complete a digital questionnaire with the assistance of educational investigators, teacher educators, and local education officers. To ensure that the participants in the survey were teachers involved in IE, only those who met the following criteria were selected: (1) Their schools had implemented IE reforms; (2) They taught students with special educational needs (SEN) in their classrooms and were expected to teach inclusively; (3) They were involved in teaching specific subjects such as Chinese, mathematics, English, etc. Teachers participated voluntarily and completed the online questionnaire investigation in July 2022. Each participant completed an online consent form before proceeding with the questionnaire. Ethics approval was obtained from the Human Research Ethics Committee of the first author’s university before data collection.

### 4.2. Demographic Information

Initially, 925 surveys were received from primary and secondary schools in Gansu province and Ningxia Hui Autonomous Region. After data cleaning, 712 survey forms were retained (response rate = 77.29%) and then used for data analysis. Demographic information on the participants is shown in Table 1. Of the participants, there were 207 males (29.1%) and 505 females (70.9%). As for their years of teaching experience, 123 (17.7%) of them had taught for no more than 5 years, 131 (18.4%) had taught from 5 to 10 years, 172 (24.2%) had taught between 11 and 20 years, and 283 (39.7%) had taught for more than 20 years. Among them, 452 (63.5%) were from primary schools and 260 (36.5%) from secondary schools.

### 4.3. Measures

#### 4.3.1. Principal Transformational Leadership

Principal transformational leadership was measured by a 9-item scale adapted from the transformational leadership scale [19]. The scale consists of three components, i.e., setting directions (3 items), developing people (3 items), and redesigning the organisation (3 items). The calculated Cronbach’s *α* for the overall PTL scale (*α* = 0.968) and its three subscales (α = 0.899, 0.957, and 0.964, respectively) showed good reliability in this study. The PTL scale also showed a satisfactory model fit (χ^2^ = 122.178, *df* = 24; RMSEA = 0.076; CFI = 0.988; TLI = 0.983; SRMR = 0.018) [66], with high factor loadings ranging from 0.778 to 0.961.

#### 4.3.2. Teachers’ Inclusive Role Identity

Teachers’ inclusive role identity was measured by a scale adapted from Callero’s (1985) role identity scale [67]. In this study, the unidimensional TIRI scale contains 4 items (α = 0.923). The CFA results showed a good model fit (χ^2^ = 2.627, *df* = 2; RMSEA = 0.021; CFI = 1.000; TLI = 0.999; SRMR = 0.004) [66], with all the factor loadings significant (*p* < 0.001; 0.787–0.935).

#### 4.3.3. Teacher Efficacy for Inclusive Practice

Teacher efficacy for inclusive practice was measured by a 10-item scale adapted from Sharma, U., T. Loreman and C. Forlin [68]. The scale consists of three dimensions: efficacy in instruction (3 items), efficacy in collaboration (3 items), and efficacy in managing behaviours (4 items). The overall TEIP scale (α = 0.910) and the three subscales (α = 0.859, 0.913, and 0.925, respectively) showed good reliability in this study. The CFA results showed a good model fit (χ^2^ = 32.826, *df* = 24; RMSEA = 0.023; CFI = 0.998; TLI = 0.997; SRMR = 0.018) [66], with all the factor loadings significant (*p* < 0.001; 0.732–0.926).

#### 4.3.4. Teachers’ Inclusive Teaching Behaviour

Teachers’ inclusive teaching behaviour was measured by a 10-item scale adapted from the scale designed by Roy and associates [69]. The scale in this study consists of three dimensions: content-oriented instructional adaptation (4 items), process-oriented instructional adaptation (3 items), and product-oriented instructional adaptation (3 items). The overall TITB scale (*α* = 0.958) and three subscales (*α* = 0.954, 0.867, and 0.903, respectively) showed good reliability. The CFA results showed a good model fit (χ^2^ = 107.564, *df* = 24; RMSEA = 0.070; CFI = 0.987; TLI = 0.981; SRMR = 0.016) [66], with all the factor loadings significant (*p* < 0.001; 0.820–0.936). 

#### 4.3.5. Control Variables

Teachers’ gender, years of teaching experience, number of SEN students taught in a classroom, and school type were selected as control variables in order to eliminate potential confounding effects that might influence the relationships among the focal variables [12,54].

### 4.4. Data Analysis

SPSS 26.0 and Mplus 8.3 software were employed to analyse the data. First, descriptive statistics and correlation analysis were conducted by SPSS. Second, confirmatory factor analysis (CFA) was conducted to examine the construct validity and distinctiveness. The measurement and confirmation of reliability were achieved by Cronbach’s alpha test of internal consistency. The average variance extracted (AVE) and composite reliability (CR) values were calculated for assessing convergent and discriminant validity. Then, structural equation modelling (SEM) was conducted to calculate the structural parameters of the latent variables. The SEM is beneficial for reducing measurement errors and calculating mediating effects in a single analytical model, leading to more precise results [70]. According to Hu, L. T. and P. M. Bentler [66], the acceptable model fit indices cutoffs were set at Tucker–Lewis index (TLI) > 0.90, comparative fit index (CFI) > 0.90, standardised root mean square residual (SRMR) < 0.06, and root mean square error of approximation (RMSEA) < 0.08. In mediation analysis, the bias-corrected bootstrapping analysis, a nonparametric resampling technique, was conducted with 5000 resamples to precisely calculate the point estimates of the indirect effects’ 95% confidence intervals [70].

Before the model analysis, normality, multicollinearity, and outliers were checked to ensure that the initial assumptions were not violated. In addition, Harman’s single-factor test was conducted to rule out possible common method bias. Since the overall variation explained (43.71%) was less than 50% when all components were loaded into one common factor, a common procedure bias did not impact our data and hence the results [71].

## 5. Results

### 5.1. Correlation Analysis

The results of the correlation analysis of the focus variables are demonstrated in Table 2.

### 5.2. Structural Equation Modelling (SEM)

**Figure 2:**
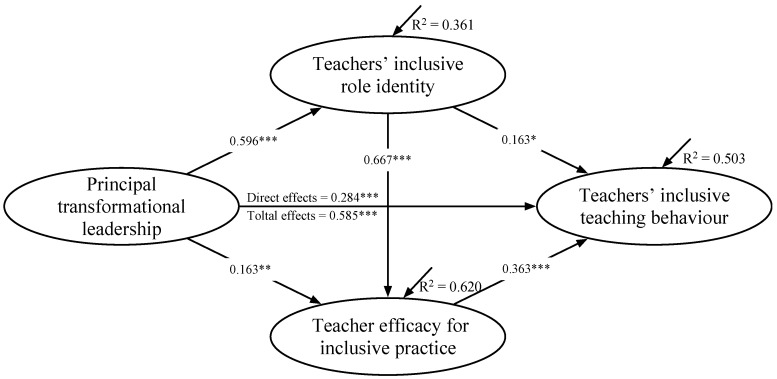
The SEM results of this study. Note. *** *p* < 0.001, ** *p* < 0.01, * *p* < 0.05.

SEM analysis was conducted to examine the relationships among PTL, TIRI, TEIP, and TITB, with control variables (i.e., gender, teaching experience, school type, and number of SEN students taught in a classroom) being considered. The results are illustrated in Figure 2. As demonstrated, the model explained a large proportion (i.e., 36.1%, 62.0%, and 50.3%) of the total variance in TIRI, TEIP, and TBTI, respectively. Furthermore, the model fit indices generated were χ^2^/*df* = 2.499, *p* < 0.001; RMSEA = 0.046; CFI = 0.967; TLI = 0.963; and SRMR = 0.047, indicating a good SEM model fit [66].

As Figure 2 illustrates, the structural portion of the SEM generated significant total influences of PTL on TBTI (*β* = 0.585, *p* < 0.001) without the inclusion of mediating variables. With the inclusion of TIRI and TEIP, PTL had positive and direct influences on TBTI (*β* = 0.284, *p* < 0.001), thus supporting the research hypothesis that PTL had positive effects on TBTI (H1). 

Furthermore, PTL had significant and positive influences on both TIRI (*β* = 0.596, *p* < 0.001) and TEIP (*β* = 0.163, *p* < 0.001). Both TIRI (*β* = 0.163, *p* < 0.001) and TEIP (*β* = 0.363, *p* < 0.001) had significant and positive influences on TBTI. TIRI also had significant and positive influences on TEIP (*β* = 0.667, *p* < 0.001).

Table 3 shows the mediating effects of TIRI and TEIP on the PTL influences on TITB. TIRI (*β* = 0.097, *p* < 0.05, 95% bootstrap CIs = [0.010, 0.189]) and TEIP (*β* = 0.059, *p* < 0.01, 95% bootstrap CIs = [0.016, 0.125]) independently mediated the influences of PTL on TITB, thereby supporting H2 and H3. Meanwhile, TIRI and TEIP sequentially mediated the relationships between PTL and TITB (*β* = 0.144, *p* < 0.001, 95% bootstrap CIs = [0.066, 0.254]). Therefore, H4 was supported. 

## 6. Discussion

This study examined the influences of principal transformational leadership on teachers’ inclusive teaching behaviour, with a particular focus on the mediating roles of teachers’ inclusive role identity and efficacy. The study yielded three major findings: (1) Principal transformational leadership had a significant direct influence on teachers’ inclusive teaching behaviour; (2) Teachers’ inclusive role identity and efficacy independently mediated the effects of principal transformational leadership on teachers’ inclusive teaching behaviour; (3) Teachers’ inclusive role identity and efficacy sequentially mediated the effects of principal transformational leadership on teachers’ inclusive teaching behaviour.

Inconsistent with the common conclusion that the effects of PTL on teaching practices are indirect [16], this study found that PTL has a significant and direct influence on TITB. Two reasons may explain the results. First, given that Oriental culture embodies high-power distance and collectivism, principals in China are considered the most potent agent to conduct school reform and influence teachers’ behaviours [72]. Therefore, PTL, which has been exanimated as an influential factor in high-power distance and collectivist cultures compared to that in the context of low-power distance and individualistic cultures [73,74], may have a more significant effect on teachers’ teaching behaviours. Second, previous research usually involved a set of mediating variables [16], while this study involved just two mediating variables. These two mediators may not be sufficient to account for the total influences of PTL on TITB.

This study reveals that teachers’ inclusive role identity positively mediated the influences of PTL on TITB, which has rarely been explored before. The results validate the critical importance of role identity as a motivating force in driving individuals to initiate behavioural actions [60,75]. In the context of promoting IE reforms, when teachers positively internalise, prioritise, and embrace their role as inclusive educators they may be motivated to adopt inclusive teaching behaviours to address the diverse learning needs of all students in their classrooms [51,76]. Additionally, the results substantiate that teachers’ inclusive role identity was significantly influenced by PTL. This is in line with identity theory [24], which posits that the social interactions between key figures generate the most impactful social information in relation to role identity [44,77]. As such, this study contributes to the literature by unravelling the mechanism of teachers’ inclusive role identity underlying the influences of PTL on TITB. 

In addition, this study also reveals that teacher efficacy for inclusive practice also significantly mediated the PTL influences on TITB. On the one hand, the results substantiate prior studies which underscored the prominent role of teacher efficacy for inclusive practice as a vital psychological strength for empowering inclusive teachers to address various challenges and constraints and achieve high-quality inclusion [12,68,78]. It can also nurture teachers’ perseverance and resilience, enabling them to continuously refine their skills, techniques, and professionalism in implementing innovative strategies for inclusive teaching [10,37]. On the other hand, the social cognitive theory denotes that teachers’ teaching behaviours can be derived from the interactions of school contextual and teacher psychological factors, where efficacy plays a central mechanism in explaining behaviour change [12,26]. This study advances the literature by further unravelling the interaction between PTL and teacher efficacy for inclusive practice in shaping the development of TITB in the context of IE reforms. Therefore, this study provides valuable insights into the mechanism of teacher efficacy for inclusive practice and how it mediates the influences of PTL on TITB.

In addition, this study reveals that PTL exerted influences on TITB through the sequential mediation of teachers’ inclusive role identity and efficacy. Specifically, the results verify teachers’ inclusive role identity as a precedent factor influencing teacher efficacy for inclusive practice. This can be attributed to the fact that the development and establishment of teachers’ inclusive role identity is conducive to the accumulation of experiences as competent inclusive educators, which represents a vital source of experiential information that strengthens teacher efficacy for inclusive practice [28,46]. That is, teachers with a strong inclusive role identity can be more prone to demonstrate commitment to the principles of inclusion and cultivate a robust belief in incorporating inclusive attitudes, knowledge, and skills into their classroom teaching [45,46]. To step further, the results elucidate that PTL as an influential school contextual influence interacted with the two teacher-level psychological factors to impact TITB. In this sense, this study made a significant contribution by addressing a research gap and providing valuable insights into a sequential mediating mechanism between PTL and TITB within the context of IE reforms.

## 7. Implications

Both teachers’ inclusive teaching behaviours and school principals’ transformational leadership practices are vital to promoting inclusion. Some practical implications can be proposed in light of the research findings.

First, the results suggest that PTL plays a salient role in facilitating teachers’ inclusive teaching behaviour amid the ongoing Chinese school reforms towards IE. Principals who take the responsibilities of implementing IE reforms are thus suggested to exercise transformational leadership behaviours, to cultivate teachers’ inspirations on IE, and motivate them to implement inclusive teaching tactics [79]. To optimise the effectiveness of PTL, it is crucial to provide training programmes specifically tailored to enhance principals’ capacity and awareness regarding promoting inclusion within schools. These programmes should help principals improve transformational leadership practices in terms of fostering inclusive values and creating an inclusive environment, providing opportunities for teacher development focused on inclusion, and reshaping school organisations to align with inclusion principles [80].

Second, this study found that teachers’ inclusive role identity and efficacy for inclusive practice independently mediated the influences of PTL on TITB. This implies that when principals assume their role as transformational leaders, they should attend to promoting teachers’ identification with their job as inclusive professionals and strengthening their beliefs in executing optimal functioning to initiate change for inclusion. Principals can strive to foster an inclusive school culture and establish high role expectations to encourage teachers to internalise inclusive missions and enhance their efficacious beliefs in implementing inclusive teaching [81]. Principals may also involve themselves in creating facilitative structures and collaboration mechanisms that assist teachers in enhancing their awareness and self-confidence in fulfilling their role as inclusive instructors [82].

In addition, this study also found that teachers’ inclusive role identity and teacher efficacy for inclusive practice sequentially mediated the PTL influences on TITB. Therefore, when enacting transformational leadership strategies to enhance the inclusive teaching behaviours of teachers, it is necessary for principals to simultaneously attend to teachers’ understanding of being inclusive professionals and their beliefs in delivering inclusive teaching. To achieve this goal, school principals and managers should involve teachers in formulating the consensus for high-quality inclusive teaching, offering practical solutions for teachers to reference in their classroom instruction, and implementing praise and feedback systems that encourage teachers’ genuine engagement in promoting inclusion.

## 8. Limitations

While this study offers valuable insights into the impact of principals’ transformational leadership on teachers’ inclusive teaching behaviour, there are some limitations to consider. Firstly, since the data were gathered in two provinces of China, this study cannot yield generalisability of the findings. Cautions should be exercised when applying the research results to other educational contexts. Secondly, the study’s cross-sectional design precludes causal inferences. Future studies may employ a longitudinal research design to examine causal relationships between the variables. Thirdly, all the data were self-reported by teachers, indicating a risk of social desirability bias and common method bias. To address this issue, it would be beneficial to collect data from additional sources (e.g., students and supervisors) and through multiple approaches (e.g., classroom observation and interviews).

## 9. Conclusions

The research, grounded in transformational leadership theory, identity theory, and social cognitive theory, advances the empirical understanding as to whether and how PTL exerts influences on TITB, which is particularly valuable against the backdrop of ongoing worldwide inclusive education reform. Echoing the emphasis of PTL in theory, policy, and practice, this study empirically found the significant and positive effects of PTL on TITB. Particularly, this study contributed to the literature by unpacking the mediating mechanisms of teachers’ inclusive role identity and teacher efficacy for inclusive practice regarding the PLT effects on TITB, echoing the call for exploring the multi-mediation and continuous mediation effects of transformational leadership on employee work performance [83]. This study thus highlights the importance of integrating transformational leadership into principals’ leadership practice and principal preparation programmes within the context of promoting inclusive education reforms in China.

## Figures and Tables

**Table 1:** Sample demographics (*n* = 712).

Demography	Variables	Frequency	Percentage
Gender	Male	207	29.1%
	Female	505	70.9%
Teaching experience (years)	0–5	123	17.7%
	5–10	131	18.4%
	10–20	172	24.2%
	Over 20	283	39.7%
Number of SEN students taught in a classroom	1–2	620	87.1%
	3 and over	92	12.9%
School type	Primary	452	63.5%
	Secondary	260	36.5%

**Table 2:** Correlation matrix.

Variables	Mean	SD	AVE	CR	PTL	TIRI	TEIP	TITB
PTL	3.653	1.259	0.848	0.981	1			
TIRI	3.802	1.246	0.755	0.925	0.594 ***	1		
TEIP	3.828	0.996	0.756	0.981	0.501 ***	0.687 ***	1	
TITB	3.940	1.081	0.796	0.981	0.559 ***	0.567 ***	0.583 ***	1

Note. *** *p* < 0.001; PTL = principal transformational leadership; TIRI = teachers’ inclusive role identity; TEIP = teacher efficacy for inclusive practice; TITB = teachers’ inclusive teaching behaviours.

**Table 3:** The mediating effects of teachers’ inclusive role identity and efficacy.

	*β*	SE	*β*/SE	95% Bootstrap CIs	
				Lower	Upper
PTL → TITB (direct effects)	0.284 ***	0.048	5.877	0.160	0.410
PTL → TIRI → TITB	0.097 *	0.045	2.167	0.010	0.189
PTL → TEIP → TITB	0.059 **	0.021	2.826	0.016	0.125
PTL → TIRI → TEIP → TITB	0.144 ***	0.035	4.103	0.066	0.254
Total indirect effects	0.301 ***	0.032	9.290	0.222	0.387
Total effects	0.585 ***	0.035	16.622	0.485	0.672

Note. Standardised coefficients are reported; * *p* < 0.05, ** *p* < 0.01, *** *p* < 0.001; PTL = principal transformational leadership; TIRI = teachers’ inclusive role identity; TEIP = teacher efficacy for inclusive practice; TITB = teachers’ inclusive teaching behaviour.

## Data Availability

The data presented in this study are available on request from the corresponding author.
